# 14-Year Epidemiologic study of *Pseudomonas aeruginosa* bloodstream infection incidence and resistance in the Veterans Health Administration system, 2009–2022

**DOI:** 10.1093/jacamr/dlae031

**Published:** 2024-03-05

**Authors:** Leila S Hojat, Brigid M Wilson, Michael J Satlin, Federico Perez, Maria F Mojica, Mendel E Singer, Robert A Bonomo, Lauren H Epstein

**Affiliations:** Division of Infectious Diseases & HIV Medicine, Department of Medicine, Case Western Reserve University, 11100 Euclid Avenue, 44106, Cleveland, OH, USA; Division of Infectious Diseases & HIV Medicine, Department of Medicine, University Hospitals Cleveland Medical Center, Cleveland, OH, USA; Division of Infectious Diseases & HIV Medicine, Department of Medicine, Case Western Reserve University, 11100 Euclid Avenue, 44106, Cleveland, OH, USA; Geriatric Research Education and Clinical Center (GRECC), The VA Northeast Ohio Healthcare System, Cleveland, OH, USA; Division of Infectious Diseases, Department of Medicine, Weill Cornell Medicine, New York, NY, USA; Division of Infectious Diseases & HIV Medicine, Department of Medicine, Case Western Reserve University, 11100 Euclid Avenue, 44106, Cleveland, OH, USA; Geriatric Research Education and Clinical Center (GRECC), The VA Northeast Ohio Healthcare System, Cleveland, OH, USA; Cleveland VAMC Center for Antimicrobial Resistance and Epidemiology (Case VA CARES), Case Western Reserve University, Cleveland, OH, USA; Research Service, VA Northeast Ohio Healthcare System, Cleveland, OH, USA; Grupo de Resistencia Antimicrobiana y Epidemiología Hospitalaria, Universidad El Bosque, Bogotá, Colombia; Department of Population and Quantitative Health Sciences, Case Western Reserve University, Cleveland, OH, USA; Division of Infectious Diseases & HIV Medicine, Department of Medicine, Case Western Reserve University, 11100 Euclid Avenue, 44106, Cleveland, OH, USA; Geriatric Research Education and Clinical Center (GRECC), The VA Northeast Ohio Healthcare System, Cleveland, OH, USA; Cleveland VAMC Center for Antimicrobial Resistance and Epidemiology (Case VA CARES), Case Western Reserve University, Cleveland, OH, USA; Research Service, VA Northeast Ohio Healthcare System, Cleveland, OH, USA; US Department of Veterans Affairs Medical Center, Emory University, Atlanta, Georgia, USA

## Abstract

**Background:**

Multidrug resistant *Pseudomonas aeruginosa* (PA) represents a serious threat to hospitalized patients. Characterizing the incidence of PA infection and degree of resistance can inform empiric treatment and preventative measures.

**Objectives:**

We sought to describe trends in incidence and resistance characteristics of PA bloodstream infections (BSI) observed within the Veterans Health Administration (VHA) system and identify factors contributing to higher observed mortality within this population.

**Methods:**

We characterized demographic and clinical features of unique patients among the VHA population presenting with their first episode of PA-BSI between 2009 and 2022 and summarized trends related to mortality and resistance phenotype based on year and geographical location. We additionally used logistic regression analysis to identify predictors of 30-day mortality among this cohort.

**Results:**

We identified 8039 PA-BSIs during the study period, 32.7% of which were hospital onset. Annual PA-BSI cases decreased by 35.8%, and resistance among all antimicrobial classes decreased during the study period, while the proportion of patients receiving early active treatment based on susceptibility testing results increased. Average 30-day mortality rate was 23.3%. Higher Charlson Comorbidity Index, higher mAPACHE score, VHA facility complexity 1b and hospital-onset cases were associated with higher mortality, and early active treatment was associated with lower mortality.

**Conclusions:**

PA-BSI resistance decreased across the VHA system during the study period. Further investigation of antimicrobial stewardship measures possibly contributing to the observed decreased resistance in this cohort and identification of measures to improve on the high mortality associated with PA-BSI in the VHA population is warranted.

## Introduction

*Pseudomonas aeruginosa* (PA) is associated with high rates of morbidity and mortality in the hospital setting. In 2017, PA was estimated to have caused 32 600 infections and 2700 deaths among hospitalized patients in the USA; from 2019 to 2020, this rate increased by 32%.^1,2^ Due to its intrinsic resistance to many therapeutic agents, treatment of infections caused by PA is challenging, and the emergence of difficult-to-treat (DTR) and XDR PA infections has further complicated treatment efforts.^3–5^ Quantifying the burden of DTR and XDR PA infections would help streamline antimicrobial stewardship efforts and inform empiric treatment in hospitalized patients. However, recent long-term trends in the rates of PA resistance have not been well described with respect to resistance phenotypes and geographical region within the USA. In this study, we aimed to identify trends in annual new PA bloodstream infection (PA-BSI) rates and resistance phenotypes among unique patients within Veterans Health Administration (VHA) hospitals. We also sought to explore the association of these trends with mortality and describe additional contributing factors to higher observed mortality.

## Materials and methods

### Population and data source

We conducted a retrospective cohort study using the nationwide VHA Corporate Data Warehouse (CDW), a repository of patient data extracted from the electronic health records of all patients within the VHA system. We included all instances of PA isolated in at least one blood culture between 1 January 2009 and 31 December 2022 among patients with at least one hospital admission since 2006. Only the first incidence of PA-BSI was included for each patient, thus each case represented a unique patient.

### Covariates and definitions

We extracted information from the CDW regarding demographics, comorbidities, vitals, basic laboratory data, mortality data, geographic location, facility complexity, case year, timing of culture relative to admission date, AST results and antimicrobial use data. Comorbid medical conditions were summarized by the Charlson Comorbidity Index as a continuous variable. The modified APACHE (mAPACHE) score, a modification of APACHE III validated within the VHA system, was calculated to determine the severity of illness based on the 48 hours following time of culture collection.^6^ Geographic location was defined by the Veterans Integrated Services Network (VISN), comprising 18 regional care systems to which all VHA facilities are mapped.^7^ Facility complexity was defined by the VHA Clinical Complexity Index that classifies facilities into five levels on the basis of volume, patient risk, teaching, research, physician specialties, size and intensive care unit facilities, ranging from 1a (most complex) to 3 (least complex).^8^ VISN configurations and facility complexity index were assigned according to classification as of October 2023. If a patient had been transferred from a lower complexity centre to a higher complexity centre, only the higher facility complexity level was included in the analysis. Timing of the index culture relative to admission date was used to classify cases as hospital onset, which was defined as cultures obtained greater than 48 hours after admission date.

We collected AST results among seven antimicrobial classes with potential activity against PA, including penicillins (piperacillin/tazobactam), cephalosporins (ceftazidime, cefepime, ceftolozane/tazobactam, ceftazidime/avibactam, cefiderocol), carbapenems (meropenem, imipenem/cilastatin, meropenem/vaborbactam, imipenem/cilastatin/relebactam, doripenem), fluoroquinolones (ciprofloxacin, levofloxacin), aminoglycosides (gentamicin, tobramycin, amikacin, plazomicin), monobactams (aztreonam) and polymyxins (polymyxin B, colistin). Susceptibility to each agent was determined by the microbiology laboratory used by each facility and was characterized as either susceptible or resistant. Isolates that were resistant or intermediate to any antimicrobial within a class prompted a designation of resistant. Susceptibility to any antimicrobial within a class in absence of any agent identified as resistant within the same class prompted a designation of susceptible. Isolates with absence of AST data for aztreonam and polymyxins were considered to be susceptible to these classes.

**Figure 1.**
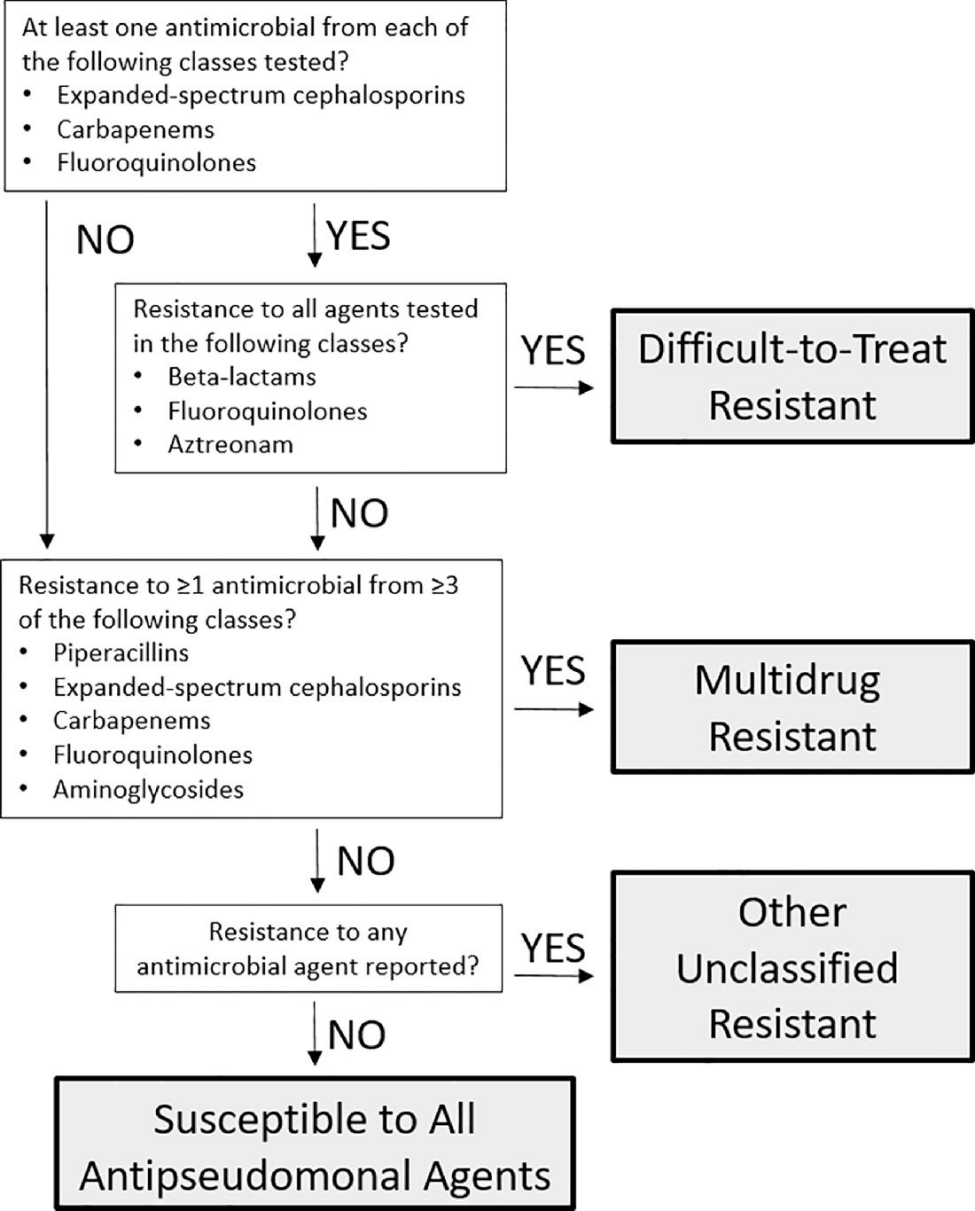
Flowchart demonstrating algorithm for classification of resistance phenotypes.

We then classified PA isolates into one of four resistance phenotypes (Figure 1): (i) difficult-to-treat resistant (DTR), (ii) multidrug resistant (MDR), (iii) other unclassified resistant (OUR) and (iv) susceptible to all antipseudomonal agents (PS). Isolates classified as DTR demonstrated *in vitro* resistance to all beta-lactam and fluoroquinolone agents, as well as aztreonam if tested.^9^ AST results for at least one antimicrobial from each of the expanded-spectrum cephalosporin, carbapenem and fluoroquinolone classes were required to designate an isolate as DTR. Isolates not meeting criteria for DTR and having *in vitro* resistance to at least one antimicrobial from at least three classes of agents including piperacillins, expanded-spectrum cephalosporins, fluoroquinolones, carbapenems and aminoglycosides were classified as MDR.^10,11^ OUR was designated for isolates with any *in vitro* resistance not meeting criteria for DTR or MDR. Isolates with *in vitro* susceptibility to all agents tested among the seven classes with potential activity against PA were classified as PS.

We additionally created an active treatment variable using antimicrobial use data in combination with AST data. Active treatment was defined as antimicrobial therapy with confirmed susceptibility to the individual PA isolate and given within two calendar days of the index culture collection date.

### Outcomes

The primary outcome was all-cause mortality at 30 days from date of collection of the index blood culture from which PA was isolated.

### Statistical analysis

We summarized the baseline characteristics for the cohort and calculated annual case rate, annual rate of resistance to individual antimicrobial classes and annual 30-day mortality rate. Total cases were further stratified by VISN, based on the location of the facility where each case was located. The proportion of total cases relative to total distinct patients with acute hospitalizations during the study period was calculated to determine whether higher case incidence could be accounted for by a higher total number of hospitalized patients within each VISN. Trends based on resistance phenotypes over the study period were additionally described.

Univariable and multivariable logistic regression were performed to determine the odds ratio (OR) and 95% confidence interval (CI) for identification of independent predictors of the primary outcome of mortality at 30 days from the date of blood culture collection. We included demographics, comorbidities, disease severity (mAPACHE), location (facility complexity, VISN), resistance phenotype and active treatment. For these analyses, we excluded patients with death occurring within two calendar days of the index culture to avoid introducing bias based on the active treatment variable and mAPACHE score. Cases with missing data were excluded from the model. We additionally performed sensitivity analyses incorporating a random intercept for facility to account for potential heterogeneity between PA-associated mortality between individual sites and excluding data from 2020 to 2022 to remove potential bias related to the COVID-19 period. Statistical significance was defined by a 95% CI not including 1. All analyses were performed in R v.4.1.2.

### Ethics

The data used in this study were collected and analysed in accordance with a protocol approved by the Institutional Review Board of the Cleveland Veterans Affairs Medical Center to study multidrug resistant organisms in hospitalized patients (VANEOHS IRB 1583880-7).

## Results

### Study population

We identified 8039 unique case-patients with PA-BSI during the study period (Table 1). Of the 7413 cases with admission data, 2423 (32.7%) were identified as hospital onset. The cohort included predominantly older, white, non-Hispanic males with multiple chronic medical issues, consistent with general characteristics of the VHA population.^12,13^ The mAPACHE score for most patients ranged between 40 and 60, with a higher range observed among patients who died within 30 days of index culture.

**Table 1.** Characteristics of the VHA population with PA*-*BSI between 2009 and 2022, stratified by 30-day all-cause mortality and higher resistance phenotypes (DTR or multidrug resistant) versus lower resistance phenotypes (OUR or susceptible to all antipseudomonal agents)

Characteristic	Mortality status at 30 days		Resistance phenotype^a^		Total (*n* = 8039)
	Survived (*n* = 6169)	Died (*n* = 1870)	DTR or MDR (*n* = 827)	OUR or PS (*n* = 6627)	
Male sex (%)	6021 (97.6)	1825 (97.6)	810 (97.9)	6465 (97.6)	7846 (97.6)
Age (median [IQR])	70 [63–79]	72 [65–80]	68 [61–77]	71 [64–79]	71 [64–79]
Race (%)					
White	4299 (69.7)	1280 (68.4)	541 (65.4)	4625 (69.8)	5579 (69.4)
Black	1418 (23.0)	427 (22.8)	214 (25.9)	1498 (22.6)	1845 (23.0)
Other	71 (1.2)	23 (1.2)	12 (1.5)	70 (1.1)	94 (1.2)
Unknown	381 (6.2)	140 (7.5)	60 (7.3)	434 (6.5)	521 (6.5)
Ethnicity (%)					
Not Hispanic or Latino	5425 (87.9)	1604 (85.8)	676 (81.7)	5805 (87.6)	7029 (87.4)
Hispanic or Latino	505 (8.2)	162 (8.7)	109 (13.2)	540 (8.1)	667 (8.3)
Other/unknown	239 (3.9)	104 (5.6)	42 (5.1)	282 (4.3)	343 (4.3)
Charlson Comorbidity Index (median [IQR])	4 [2–6]	5 [3–7]	5 [3–7]	4 [2–6]	4 [2–6]
mAPACHE score (median [IQR])^a^	48 [38–60]	67 [54–80]	56 [41–65]	53 [40–60]	51 [40–64]
Facility complexity (%)^a^					
1a	3417 (56.6)	1066 (57.5)	530 (65.6)	3663 (56.3)	4483 (56.8)
1b	1172 (19.4)	391 (21.1)	147 (18.2)	1332 (20.5)	1563 (19.8)
1c	888 (14.7)	271 (14.6)	85 (10.5)	968 (14.9)	1159 (14.7)
2	395 (6.5)	101 (5.4)	34 (4.2)	411 (6.3)	496 (6.3)
3	166 (2.7)	26 (1.4)	12 (1.5)	133 (2.0)	192 (2.4)
Resistance phenotype (%)^a^					
DTR	201 (3.5)	102 (6.0)	—	—	303 (4.1)
MDR	396 (6.9)	128 (7.5)	—	—	524 (7.0)
OUR	1479 (25.7)	422 (24.7)	—	—	1901 (63.4)
PS	3672 (63.9)	1054 (61.8)	—	—	4726 (63.4)
Active treatment (%)^a^	4867 (86.0)	1327 (81.8)	349 (44.7)	5570 (90.6)	6194 (85.1)
Hospital onset (%)^a^	1636 (29.3)	787 (43.2)	476 (63.9)	1766 (28.8)	2423 (32.7)

Abbreviations: DTR, difficult-to-treat resistant; OUR, other unclassified resistant; PS, susceptible to all antipseudomonal agents; IQR, interquartile range.

^a^Resistance phenotype missing for 585 isolates, mAPACHE score missing for 1711 isolates, facility complexity missing for 146 isolates, active treatment missing for 757 isolates and location of onset missing for 626 isolates.

### Facility and location characteristics

**Figure 2.**
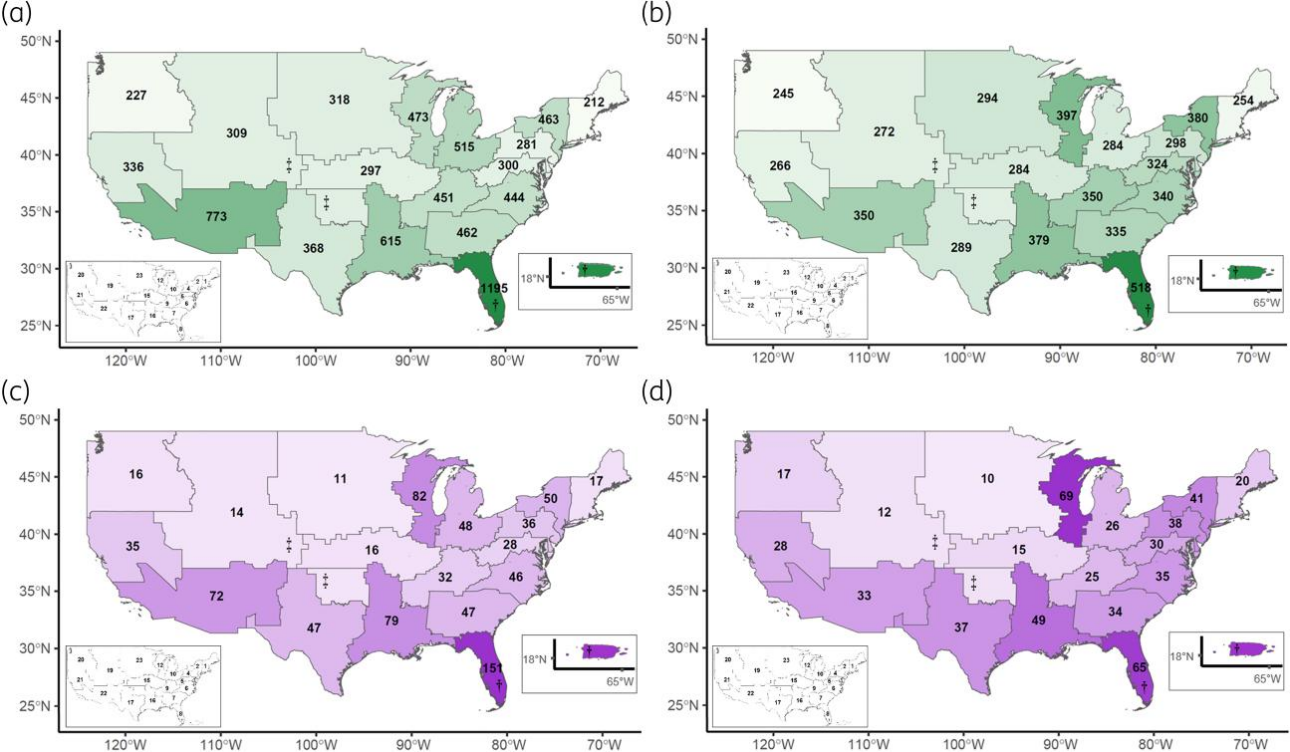
VISN map with total number of PA*-*BSI cases (a), total cases per 100 000 unique hospitalized patients (b), total multidrug resistant (MDR) or difficult-to-treat (DTR) resistant cases (c), and total MDR or DTR cases per 100 000 unique hospitalized patients (d). Darker shades indicate a higher number or proportion of cases. Dagger symbols († and ‡) indicate areas that correspond to a non-contiguous area within the same VISN. The small inset maps provides VISN number references.

Most case-patients either initially presented or were transferred to high complexity (1a) centres (Table 1). The total number of cases during the study period widely varied by VISN (Figure 2a). The VISNs with the highest total number of cases included: VISN 8, which includes Puerto Rico and most of Florida (*n* = 1195, 14.9%); VISN 22, which includes southern California, Arizona and most of New Mexico (*n* = 773, 9.6%); and VISN 16, including Louisiana, most of Arkansas and Mississippi, and eastern Texas (*n* = 615, 7.7%). These three areas accounted for 32.1% of cases. The VISNs with the lowest number of cases were VISN 1 (*n* = 212, 2.6%), VISN 20 (*n* = 227, 2.8%) and VISN 4 (*n* = 281, 3.5%), representing the US Northeast, US Northwest and most of Pennsylvania, respectively. VISN 8 maintained the largest number of patients after correcting for number of unique hospitalized patients per VISN, although the variation between the rest of the VISNs was less pronounced after accounting for this factor (Figure 2b).

### Total cases and resistance trends

**Figure 3.**
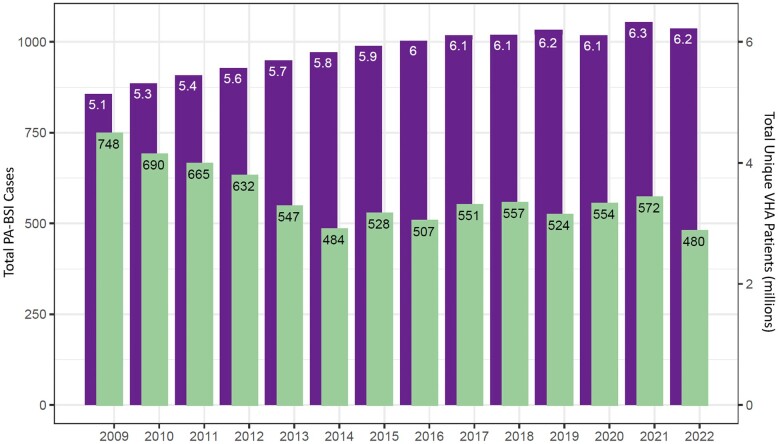
Annual trend of number of PA*-*BSI cases in the VHA system (green bars, left y-axis) and total unique VHA patients per fiscal year in millions, rescaled for comparison (purple bars, right y-axis), 2009–2022. Fiscal year begins 1 October and ends 30 September, designated by calendar year in which it ends.

Annual PA-BSI cases decreased by 35.8% overall during the study period between 2009 and 2022, for an average annual decrease of 2.6%. This decline primarily occurred from 2009 to 2014 when the annual number of PA-BSI cases steadily decreased from 748 to 484. No distinct trend was observed between 2014 and 2022, with annual cases ranging between approximately 480 and 580 (Figure 3). This is relative to the total number of unique patients seeking care at a VHA facility each year, which increased annually by an average of 1.3% between 2009 and 2022.^14^

**Figure 4.**
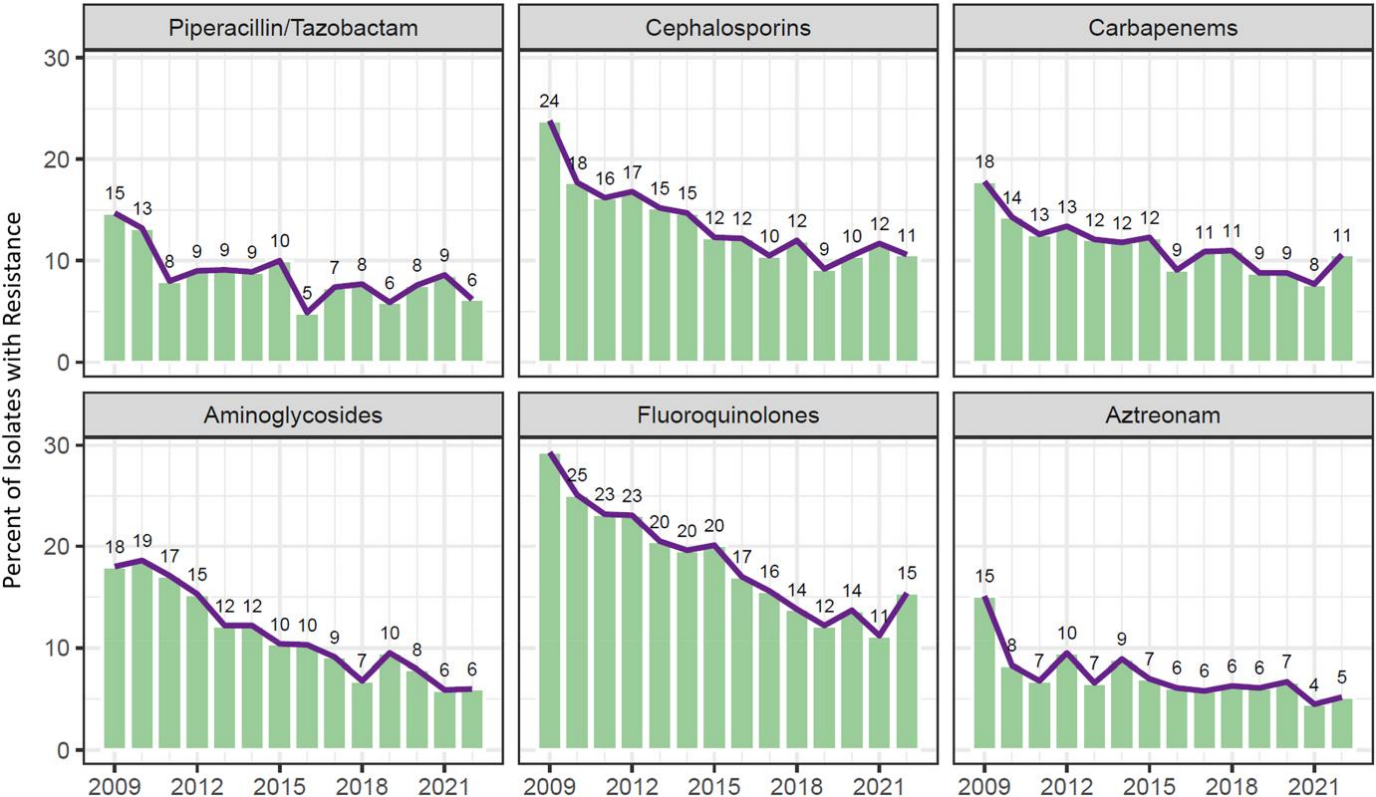
Annual trend of the percentage of PA*-*BSI cases in the VHA system 2009–2022 demonstrating *in vitro* antibiotic resistance stratified by individual agent or class: piperacillin/tazobactam, cephalosporins (ceftazidime, cefepime), carbapenems (meropenem, imipenem/cilastatin, doripenem), fluoroquinolones (ciprofloxacin, levofloxacin), aminoglycosides (gentamicin, tobramycin, amikacin) and aztreonam. Susceptibility is reported to the extent that data were available; missing data included piperacillin/tazobactam (10.6%), cephalosporins (3.2%), carbapenems (5.1%), fluoroquinolones (3.9%), aminoglycosides (2.6%) and aztreonam (69.9%). Polymyxin (polymyxin B, colistin) data were missing in 96.7% of cases; reported resistance was 2.7% among isolates with available data (not shown).

The percentage of resistant PA-BSIs decreased across all classes during the study period (Figure 4). Less than 5% of isolates had polymyxin data reported; among the 264 cases with available AST data, resistance was 2.7% (not shown).

**Figure 5.**
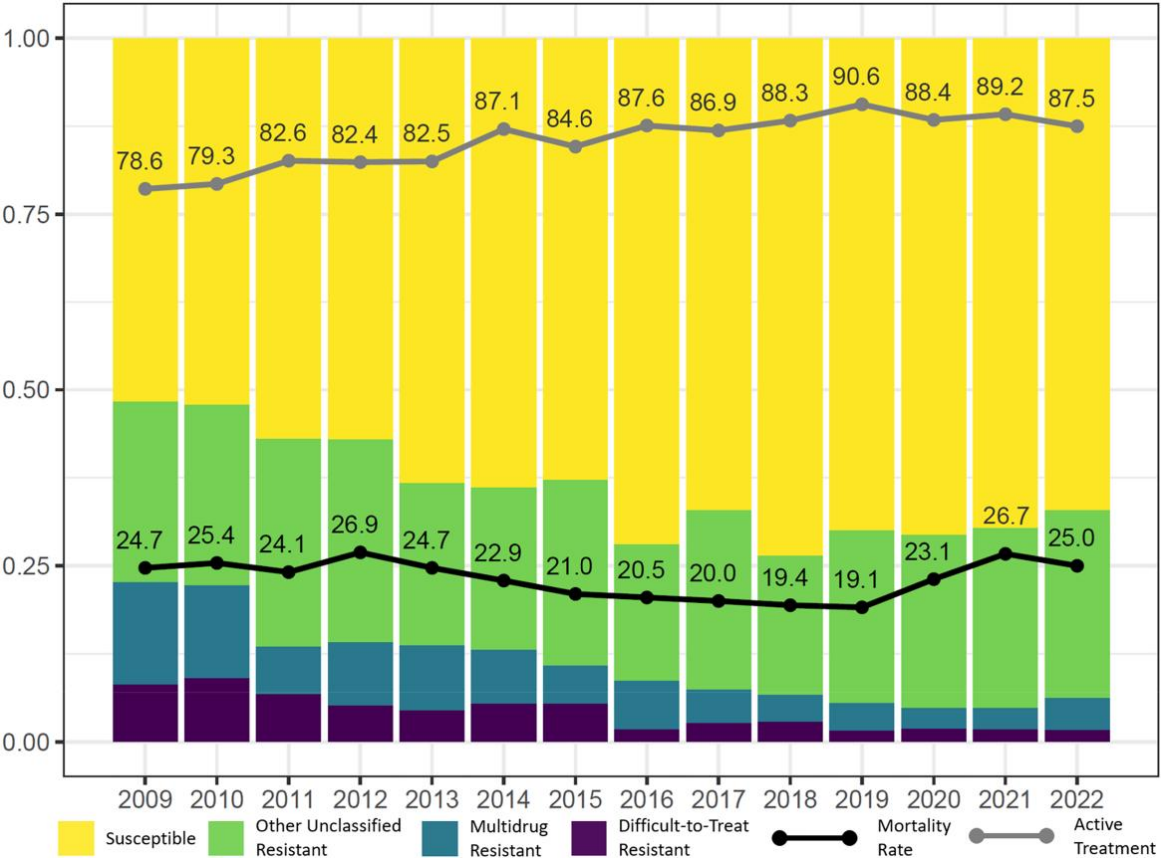
Annual trend in proportion of PA*-*BSI cases in the VHA system 2009–2022 demonstrating annual resistance phenotype distribution (stacked bar chart), annual mortality rate (black line) and proportion of cases receiving active treatment within 2 days of index culture collection date (grey line).

AST phenotypes varied among VISNs, with the largest number of MDR or DTR cases in VISN 8 (*n* = 151, 18.3%) and VISN 12 (*n* = 82, 9.9%); VISN 8 as above includes FL and PR and VISN 12 corresponds mainly to parts of Illinois (including Chicago) and Wisconsin (Figure 2c). Of note, 50.3% (*n* = 76) of the resistant isolates in VISN 8 were DTR phenotype, of which 61.8% (*n* = 47) were located in Puerto Rico, relative to only 18.3% (*n* = 15) DTR phenotype in VISN 12. After normalizing for number of hospitalized patients, VISNs 8 and 12 had comparable MDR or DTR resistance to each other and higher resistance relative to other VISNs, while resistance rates were lowest in northwest and central VISNs (Figure 2d). Resistance based on phenotype also varied across race (higher in black patients), ethnicity (higher in Hispanic or Latino patients) and facility complexity (higher in 1a centres). With respect to annual trends in phenotype distribution, the proportion of cases with PS phenotype increased and the proportion with MDR and DTR phenotypes decreased from 2009 to approximately 2018, after which the distribution of phenotypes had limited change (Figure 5). With this decrease in resistant phenotypes, we observed an increase in active treatment rates, from 78.6% in 2009 to a high of 90.6% in 2019. The average mortality rate during the study period was 23.3%, decreasing from 24.7% in 2009 to a low of 19.1% in 2019. Mortality increased between 2020 and 2022, with an average mortality during this period of 25.0%.

### Outcomes

In the univariable analysis, higher age, unknown ethnicity (relative to not Hispanic or Latino ethnicity), higher Charlson Comorbidity Index, higher mAPACHE score, DTR or MDR resistance phenotypes (relative to PS phenotype) and hospital-onset infections were associated with higher odds of all-cause 30-day mortality (Table 2). Facility complexity 3 (relative to 1a) and active treatment within 2 days were associated with lower mortality. Relative to VISN 8, which included the highest number of cases, only VISN 22, which had the second highest number of cases, was associated with higher mortality (OR 1.31; CI 1.02–1.67; not shown). In the multivariable analysis, higher Charlson Comorbidity Index (OR 1.04; CI 1.01–1.08), higher mAPACHE score (OR 1.05; 95% CI 1.04–1.06), facility complexity 1b versus 1a (OR 1.31; 95% CI 1.06–1.60) and hospital-onset infections (OR 1.77, 95% CI 1.48–2.11) were associated with higher odds of all-cause 30-day mortality, while active treatment within 2 days was associated with lower mortality (OR 0.75; 95% CI 0.59–0.97). Including VISN as a covariate did not improve model fit and VISN was not included in the final model. In a sensitivity analysis in which cases between 2020 and 2022 were excluded, only mAPACHE score, facility complexity 1b and hospital-onset infections were associated with higher mortality; active treatment had non-statistically significant protective effect on mortality (OR 0.77; 95% CI 0.58–1.03). In a second sensitivity analysis in which facility was included as a random effect variable, no notable shifts in the magnitude or direction of the modelled effects were observed, with or without exclusion of 2020–2022 cases (chi-square *P* = 0.43).

**Table 2.** Unadjusted and adjusted analyses for predictors of 30-day mortality in patients with PA*-*BSI in the VHA system 2009 to 2022

Predictor	30-Day mortality			
	Unadjusted		Adjusted^a^	
	OR	95% CI	OR	95% CI
Male sex	1.11	(0.74–1.73)	0.86	(0.49–1.64)
Age in years	**1**.**02**	**(1.01–1.02)**	1.01	(1.00–1.01)
Race (reference = white)				
Black	1.02	(0.88–1.19)	0.94	(0.76–1.14)
Other	0.89	(0.46–1.58)	0.58	(0.19–1.43)
Unknown	1.28	(1.00–1.62)	1.28	(0.88–1.84)
Ethnicity (reference = not Hispanic or Latino)				
Hispanic or Latino	1.02	(0.81–1.27)	0.98	(0.73–1.31)
Other/Unknown	**1**.**54**	**(1.16–2.01)**	1.1	(0.71–1.66)
Charlson Comorbidity Index	**1**.**13**	**(1.10–1.15)**	**1**.**04**	**(1.01–1.08)**
mAPACHE score	**1**.**05**	**(1.05–1.06)**	**1**.**05**	**(1.04–1.06)**
Facility complexity (reference = 1a)				
1b	1.15	(0.98–1.34)	**1**.**31**	**(1.06–1.60)**
1c	0.98	(0.82–1.18)	1.12	(0.88–1.42)
2	0.84	(0.63–1.09)	1.03	(0.71–1.47)
3	**0**.**55**	**(0.33–0.88)**	1.30	(0.68–2.32)
Resistance phenotype (reference = PS)				
DTR	**1**.**85**	**(1.38–2.45)**	1.20	(0.76–1.87)
MDR	**1**.**33**	**(1.04–1.68)**	1.08	(0.77–1.49)
OUR	1.10	(0.95–1.28)	1.01	(0.84–1.23)
Active treatment	**0**.**83**	**(0.70–0.98)**	**0**.**75**	**(0.59–0.97)**
Hospital onset	**1**.**81**	**(1.59–2.05)**	**1**.**77**	**(1.48–2.11)**

30-day mortality represented all-cause mortality by day 30 from blood culture collection. Values in bold represent statistically significant results

Abbreviations: PS, susceptible to all antipseudomonal agents; DTR, difficult-to-treat resistant; OUR, other unclassified resistant.

^a^Adjusted for sex, age, race, ethnicity, Charlson Comorbidity Index, mAPACHE score, facility complexity, resistance phenotype, active treatment and location of onset.

## Discussion

The threat of increasing rates of multidrug resistant infections in hospitalized patients is a major concern that has mobilized recent antimicrobial stewardship and infection prevention efforts. PA is one of the most common infectious pathogens globally and demonstrates complex resistance patterns amenable to limited treatment options.^15,16^ In this context, the findings of our study are encouraging. We observed an average annual 2.6% decrease in total number of unique PA-BSI patient-cases, despite an increase in the total number of unique VHA patients per year during the same period. We also observed an overall decrease in resistance to individual antipseudomonal agents as well as decrease in rates of MDR and DTR among PA-BSI.

Our findings highlight several key features related to PA-BSI epidemiology across the VHA system in the USA. First, both the incidence, resistance and mortality associated with PA-BSI have generally decreased across the VHA system since 2009, consistent with findings from previous VHA studies performed between 2003 and 2014.^17–19^ This is also consistent with national trends reported by the CDC, showing decreasing PA incidence between 2012 and 2017. CDC reported a reverse in this trend between 2019 and 2020; however, this trend was not observed across the VHA system during the same time frame.^1,2^ While the decline in mortality was likely multifactorial, broader antimicrobial susceptibility likely was a major contributing factor, given that active antipseudomonal treatment was an important protective factor in our study. In this context, it is worthwhile to recognize that the VHA mandated hospitals to establish an antimicrobial stewardship programme well before it became a Centers for Medicare and Medicaid Services requirement.^20^ This requirement led to the development of a robust antimicrobial stewardship network within the VHA system. Moreover, previous studies have identified an association between infection prevention programmes established throughout the VHA system and decreases in Gram-negative BSI.^21,22^ Although only a third of the cases included in our study were hospital onset, mortality was approximately 80% higher in this group. Thus, any impact on outcomes in hospital-onset cases would have substantial clinical impact. Mortality abruptly increased from 2019 to 2022, corresponding to the onset of the COVID-19 pandemic, which may have impacted by delays in recognition or treatment of PA-BSI, diversion of antimicrobial stewardship resources or concomitant COVID-19 illness.^23,24^

Second, geographical factors played an impactful role in PA-BSI epidemiology with the number of PA-BSI cases varying widely between VISNs. Variation between VISNs was considerably lower after correction for number of hospitalizations, although VISN 8 retained the highest case rate. VISN 8 also had the largest share of DTR or MDR cases, which was driven by a high rate of DTR cases in Puerto Rico. Puerto Rico, although often excluded from or combined with other regions in studies, was also previously recognized in the VHA system as having over twice the odds of multidrug resistance in Gram-negative isolates among veterans with spinal cord injuries compared to other locations.^25^ These findings could potentially be explained by warmer climate, which has been linked to increased antimicrobial resistance, particularly in the context of climate change,^26–28^ as well as to a higher rate of Gram-negative BSI.^29,30^ Climate-related phenomena, however, would not account for the higher resistance observed in VISN 12 after correction for hospitalizations, which could instead be related to local factors such as patient-related characteristics or infection prevention and antimicrobial stewardship efforts to mitigate such resistance. Local factors could also account for differences observed based on facility complexity, which warrants further observation. Reassignment of facility complexity levels over the study period could have been another factor that influenced these findings.

Third, while PA resistance may be decreasing, patients who nevertheless develop DTR or MDR PA-BSI may be at higher risk for mortality based on the respective 85% and 33% higher odds of 30-day mortality observed among these phenotypes in the unadjusted analysis. This observation is substantiated by many previous studies that have identified worse outcomes in patients with more resistant PA isolates.^3,31,32^ More complex resistance phenotypes, however, were not associated with higher mortality after adjustment for other factors such as active treatment, which had a protective effect in our cohort as well as in previous studies.^33^ This is important in context of only 45% of patients with DTR or MDR resistance receiving active treatment within 2 days. Of note, when excluding cases between 2020 and 2022, active treatment was not observed to be protective against mortality, potentially due to the overall lower mortality without inclusion of these cases causing the analysis to be underpowered to demonstrate this observation.

Finally, and particularly important to the VHA population, higher Charlson Comorbidity Index scores were associated with higher mortality, although this did not persist after the exclusion of 2020–2022 cases. Multimorbidity is prevalent among veterans relative to non-veterans of the same age range or sex.^34–37^ At the same time, it is well recognized that PA is associated with higher mortality and greater resistance in individuals with multimorbidity.^38–40^ This highlights the vulnerability of VHA patients related to PA infection and demonstrates the importance of further investigation of this pathogen within the VHA system.

Our study has several limitations. Regarding the trends described over the study period, theories may be offered as potential explanations, but causation is unable to be determined. Only the first case identified for each individual patient was included, and facilities may have served as referral centres for cases first identified in other states, both of which may have distorted the observed epidemiologic trends. Comparisons to the total veteran population must be considered in the context of only some veterans seeking care within VHA facilities, although the estimated population of veterans using benefits (including healthcare) increased over the same period in which we observed VHA PA-BSI cases to decrease.^14^ Resistance trends were dependent on the microbiology data available in the CDW that may have been incomplete, and laboratory methods or tested agents may have varied between different facilities or within the same facility between different years. The Clinical Laboratory and Standards Institute breakpoints for several antipseudomonal agents were lowered during the study period, although this would have been expected to lead to more resistance over time rather than our observation of less resistance.^41^ We did not consider non-bloodstream isolates such as respiratory due to the difficulty distinguishing between true infection and colonization in this setting, although non-bloodstream isolates may have greater resistance. We collected and analysed data believed to be relevant to PA-associated outcomes, however, several other important factors were not available within our dataset such as source of infection, intensive care unit status or concomitant infections including COVID-19. Moreover, our model may have been affected by missing data, which we expect would have skewed the data towards less severe outcomes. Mortality data may also have been affected by transfers to other facilities or discharges. Additionally, the VHA system serves a unique population that may not be fully representative of the entire population of the country, nor does it reflect global trends.

Despite these limitations, our study has several major strengths. With the inclusion of more than 8000 cases over a 14-year period, this represents one of the largest longitudinal studies published related to PA-BSI in any population. Evaluation of the epidemiology of PA within the VHA system is particularly worthwhile relative to other surveillance programmes that do not include the extensive corresponding clinical data available in the CDW. Furthermore, the data in this study were representative of the entire continental USA and Puerto Rico, which revealed several important geographical trends as previously mentioned. Our study has also provided a foundation for future investigations. While investigations of the impact of VHA infection prevention efforts on antimicrobial resistance have been initiated, it would be worthwhile to have more comprehensive analysis of the effects of antimicrobial stewardship on BSI particularly for PA, also in context of geographical region and facility characteristics. The observed protective effect of active treatment on mortality in this study suggests there may be a role for development of risk stratification tools for prediction of antimicrobial-resistant pathogens. Finally, the vulnerability of the VHA population and its greater than 20% mortality rate associated with PA-BSI warrants exploration of potential measures to improve outcomes related to this infection.

### Conclusion

Annual PA-BSI rate decreased overall by 36% and resistance decreased for all categories and resistance phenotypes in the VHA system between 2009 and 2022. All-cause 30-day mortality also decreased until 2019 but increased since 2020. Mortality was associated with higher disease severity, multimorbidity, within-hospital transmission and failure to initiate early active treatment. Further investigation into modifiable factors contributing to improved outcomes related to PA-BSI is necessary.

## Transparency declarations

F.P. reports receiving grant funding and research support from Accelerate, Merck and Pfizer. M.J.S. reports contract payments to his institution from Merck, BioFire Diagnostics, Affinity Biosensors and SNIPRBiome; personal consulting fees from Shionogi, and data and safety monitoring board participation for AbbVie. R.A.B. reports receiving grant funding and research support from NIH, VA, VenatoRx, Merck, Wockhardt, Entasis and the Cystic Fibrosis Foundation. All other authors report no potential conflicts.
