# Correction to: 14-Year Epidemiologic study of *Pseudomonas aeruginosa* bloodstream infection incidence and resistance in the Veterans Health Administration system, 2009–2022

**DOI:** 10.1093/jacamr/dlae084

**Published:** 2024-05-22

This is a correction to: Leila S Hojat, Brigid M Wilson, Michael J Satlin, Federico Perez, Maria F Mojica, Mendel E Singer, Robert A Bonomo, Lauren H Epstein, 14-Year Epidemiologic study of *Pseudomonas aeruginosa* bloodstream infection incidence and resistance in the Veterans Health Administration system, 2009–2022, *JAC-Antimicrobial Resistance*, Volume 6, Issue 2, April 2024, https://doi.org/10.1093/jacamr/dlae031

In the originally published version of the manuscript, there were errors in Figure 3. The left and right y-axes labels have been corrected. The left y-axis label has been changed from “Resistance Phenotype Proportion” to “Total PA-BSI Cases” and the right y-axis has been changed from “Mortality Rate” to “Total Unique VHA Patients (millions)”. (PA-BSI and VHA abbreviations are already defined in Figure 3's caption.)

Figure 3 should read:

**Figure:**
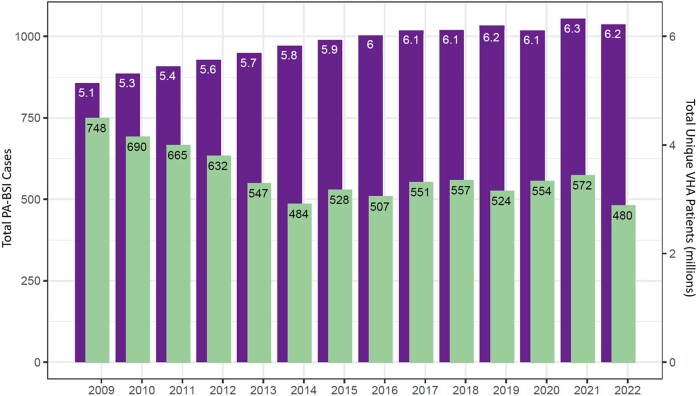


instead of:

**Figure:**
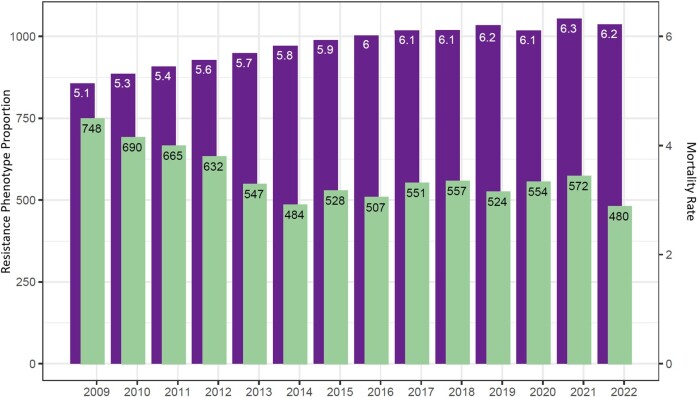


The emendation has been made to the article.

